# P-1936. Antifungal prophylaxis with echinocandin in high-risk liver transplant: a single-centre experience

**DOI:** 10.1093/ofid/ofaf695.2104

**Published:** 2026-01-11

**Authors:** Andrea Lombardi, Giulia Viero, Barbara Antonelli, Clara Dibenedetto, Vittorio Scaravilli, Luca Del Prete, Giulia Renisi, Laura Alagna, Anna Celotti, Giacomo Grasselli, Pietro Lampertico, Cristiano Quintini, Alessandra Bandera

**Affiliations:** IRCCS Ca Granda Ospedale Maggiore Policlinico, Milan, Lombardia, Italy; Università degli Studi di Milano, Milano, Lombardia, Italy; Fondazione IRCCS Ca' Granda Ospedale Maggiore Policlinico di Milano, Milano, Lombardia, Italy; SC Gastroenterologia ed Epatologia, Fondazione IRCCS Ospedale Maggiore Policlinico, Milano, Milano, Lombardia, Italy; 5- SC Anestesia e Terapia Intensiva Adulti, Fondazione IRCCS Ospedale Maggiore Policlinico, Milano, Milano, Lombardia, Italy; SC Chirurgia Generale - Trapianti di Fegato, Fondazione IRCCS Ospedale Maggiore Policlinico, Milano, Milano, Lombardia, Italy; 1- SC Malattie Infettive, Fondazione IRCCS Ospedale Maggiore Policlinico, Milano, Milano, Lombardia, Italy; 1- SC Malattie Infettive, Fondazione IRCCS Ospedale Maggiore Policlinico, Milano, Milano, Lombardia, Italy; SC Malattie Infettive, Fondazione IRCCS Ospedale Maggiore Policlinico, Milano, Milano, Lombardia, Italy; SC Anestesia e Terapia Intensiva Adulti, Fondazione IRCCS Ospedale Maggiore Policlinico, Milano, Milano, Lombardia, Italy; CRC “A. M. and A. Migliavacca” Center for Liver Disease, Department of Pathophysiology and Transplantation, University of Milan, Milan, Lombardia, Italy; SC Chirurgia Generale - Trapianti di Fegato, Fondazione IRCCS Ospedale Maggiore Policlinico, Milano, Milano, Lombardia, Italy; SC Malattie Infettive, Fondazione IRCCS Ospedale Maggiore Policlinico, Milano, Milano, Lombardia, Italy

## Abstract

**Background:**

Liver transplantation (LTx) is the definitive treatment for end-stage liver disease, but it carries a high risk of post-operative infections, particularly invasive fungal infections (IFIs). IFIs contribute to morbidity and mortality among LTx recipients, especially in high-risk patients. Current guidelines recommend targeted antifungal prophylaxis in patients with defined risk factors, but optimal strategies are still debated.

**Methods:**

This retrospective, single-center study evaluated the postoperative course of 286 adult LTx recipients at Fondazione IRCCS Ca’ Granda Ospedale Maggiore Policlinico in Milan, Italy, from January 2017 to September 2023. Patients were stratified into high-risk and non-high-risk groups based on institutional criteria, and a 14-day targeted antifungal prophylaxis regimen with echinocandin (primarily caspofungin) was administered to high-risk patients. The primary outcome was the incidence of IFIs within 30 days post-transplant. Secondary endpoints included identification of fungal species, antifungal susceptibility, and associated risk factors for IFI development.

**Results:**

Among the 286 LTx recipients, 29.72% (85/286) were classified as high-risk. The 30-day incidence of IFIs was 4.19%. High-risk patients had a higher IFI incidence (9.41% vs. 1.99%, p=0.0077), primarily driven by invasive mould infections (IMIs). In contrast, invasive Candida infections (ICIs) were less frequent and effectively prevented by caspofungin prophylaxis. Seven of eight IFIs in the high-risk group occurred during prophylaxis, classifying them as breakthrough IFIs. Risk factors associated with IFI development included diabetes (p=0.0075), MELD score >30 (p=0.0327), prolonged hospitalization ( >20 days, p=0.0347), and extended antibiotic exposure ( >10 days, p=0.0109).

**Conclusion:**

We highlighted a significant association between high-risk status and IFI development in LTx recipients despite targeted caspofungin prophylaxis. Caspofungin showed limited efficacy against IMIs, which were likely already present at the time of transplantation. These findings emphasize the need for improved early diagnostic strategies to optimize antifungal prophylaxis in high-risk LTx recipients.

**Disclosures:**

Pietro Lampertico, MD, AbbVie: Speaking/teaching fees/advisory committee/review panel|Aligos Therapeutics: Speaking/teaching fees/advisory committee/review panel|Alnylam Pharmaceuticals: Speaking/teaching fees/advisory committee/review panel|Antios Therapeutics: Speaking/teaching fees/advisory committee/review panel|Arrowhead Pharmaceuticals: Speaking/teaching fees/advisory committee/review panel|Bristol Myers Squibb: Speaking/teaching fees/advisory committee/review panel|Eiger Biopharmaceuticals: Speaking/teaching fees/advisory committee/review panel|Gilead Sciences, Inc.: Speaking/teaching fees/advisory committee/review panel|GSK: Speaking/teaching fees/advisory committee/review panel|Janssen: Speaking/teaching fees/advisory committee/review panel|Merck Sharp & Dohme: Speaking/teaching fees/advisory committee/review panel|MYR GmbH: Speaking/teaching fees/advisory committee/review panel|Roche: Speaking/teaching fees/advisory committee/review panel|Spring Bank Pharmaceuticals: Speaking/teaching fees/advisory committee/review panel

